# Physical activity in adults with epilepsy: clinical aspects and relationship with cognition and quality of life

**DOI:** 10.1590/1980-5764-DN-2022-0107

**Published:** 2023-07-24

**Authors:** Glória Maria de Almeida Souza Tedrus, Vania Aparecida Leandro-Merhi

**Affiliations:** 1 Pontífica Universidade Católica de Campinas, Programa de Pós-Graduação em Ciências da Saúde, Campinas SP, Brazil.

**Keywords:** Physical Exercise, Epilepsy, Cognition, Quality of Life, Exercício Físico, Epilepsia, Cognição, Qualidade de Vida

## Abstract

There are several factors associated with lower participation in regular physical activity (PA) in adult patients with epilepsy (PWEs).

**Objective:**

To assess the relationship between the regular practice of PA with clinical and cognitive variables and quality of life (QoL) in PWEs.

**Methods:**

Habitual Physical Activity Questionnaire (HPAQ) was related to clinical variables, scores on the Mini-Mental State Examination (MMSE), on the Brief Cognitive Battery-Edu (BCB-Edu), on the Satisfaction Scale for Physical Activity (SSPA), and on the Quality of Life in Epilepsy Inventory (QOLIE)-31 in 60 PWEs, with a significance level of p<0.05.

**Results:**

The PWEs had a mean age of 42.4±13.6 years, 50% of whom were female. Longer length of epilepsy correlated with lower PA in leisure time (Pearson correlation [r]= -0.276; p-value [p]=0.036). The occupational physical activity scores of the HPAQ correlated positively with perception (r=0.300; p=0.021), memory (r=0.381; p=0.003), semantic verbal fluency test (SVF) (r=0.427; p=0.001), and with the total score in the MMSE (r=0.327; p=0.012). The total HPAQ score correlated with the SVF (r=0.336; p=0.009) and with the MMSE (r=0.254; p=0.049). There was no correlation among the QOLIE–31, the HPAQ, and the SSPA.

**Conclusions:**

Longer duration of epilepsy was associated with the lower practice of PA. Physical activity was associated with better performance in aspects of cognition. There was no relationship between QoL and practice and satisfaction with PA, suggesting different psychosocial aspects involved.

## RESUMO

Existem vários fatores associados à menor participação em atividade física regular (AF) em pacientes adultos com epilepsia (PCE).

**Objetivo:**

Avaliar em PCEs a relação entre a prática regular de AF com as variáveis clínicas e cognitivas e com a qualidade de vida (QV).

**Métodos:**

Foi relacionado o *Habitual Physical Activity Questionnaire* (HPAQ) com as variáveis clínicas, escores no *Mini-Mental State Examination* (MMSE), no *Brief Cognitive Battery-Edu* (BCB-Edu), Satisfaction Scale for Physical Activity (SSPA) e no *Quality of Life in Epilepsy Inventory* (QOLIE–31) de 60 PCE, com nível de significância p<0,05.

**Resultados:**

Idade média 42,4±13,6 anos, 50% do sexo feminino. O maior tempo de epilepsia correlacionou-se com a menor prática de AF no lazer (Correlação de Person, r= -0,276; p=0,036). Escores da AF ocupacional do HPAQ correlacionaram-se positivamente com a percepção (r=0,300; p=0,021) e a memória (r=0,381; p=0,003) no teste de fluência verbal (SVF); (r=0,427; p=0,001) e com o escore total no MMSE (r=0,327; p=0,012). O escore total do HPAQ correlacionou-se com o SVF (r=0,336; p=0,009) e com o MMSE (r=0,254; p=0,049). Não houve correlação entre o QOLIE–31 com o HPAQ e a SSPA.

**Conclusão:**

A maior duração da epilepsia associou-se com a menor prática de AF. A AF associou-se com o melhor desempenho em aspectos da cognição. Não houve relação entre QV com a prática e a satisfação e a AF, sugerindo diferentes aspectos psicossociais envolvidos.

## INTRODUCTION

Adult people with epilepsy (PWEs) are considered more sedentary and do not engage in physical activity (PA) at the recommended intensity or frequency compared to individuals with other chronic diseases or to the general population in distinct cultural contexts^1–4^. In the last decade, the International League Against Epilepsy (ILAE) reinforced the orientation of the importance of sports and recreation for individuals with epilepsy^5^. PA is defined as any bodily movement produced by skeletal muscles that require energy expenditure^6^.

The physiological benefits of PA in different patterns of intensity and frequency are well documented in the literature^6–8^. However, the consistency and magnitude of the effects of PA on cognitive functions continue to be debated and are not yet fully understood, which indicates the complexity of the neurophysiological process involved. The positive repercussions on the performance of domains of attention, memory and executive function due to the practice of PA are related to functional and structural brain changes resulting from neuroplasticity and the release of neurotransmitters and neurotrophic factors, among others^9–12^.

Sedentary behavior is associated with low cognitive performance; however the complex interaction between PA and cognition is still unclear despite studies in different series^13^. Nevertheless, pieces of evidence to sustain these findings in epilepsy are still scarce. Thus, we hypothesized that there is a positive association between cognitive performance and PA in PWEs.

The aim of this study was to assess regular PA and the relationship with cognitive and clinical variables, satisfaction with PA practice and quality of life (QoL) in PWEs.

## METHODS

This is a cross-sectional study to assess individuals diagnosed with epilepsy treated at the clinical neurology outpatient service of the University Hospital of the Pontifical Catholic University of Campinas, Brazil, from July to December 2019. The inclusion criteria were: having been diagnosed with epilepsy for at least two years, according to the ILAE criteria^14^; being over 18 years old; neurosurgically naive; and regularly using antiseizure medications (ASMs). In addition, a limit of at least three years of formal schooling was established for inclusion in the study.

The exclusion criteria were: the presence of musculoskeletal disorders; cardiovascular diseases; and moderate or severe cognitive deficits that prevented them from regularly practicing PA.

A control group (CG) was constituted, composed of subjects with a history of neurological or other chronic disorders and similar in terms of age, sex, education, and socioeconomic level. All assessments were performed individually, in a quiet and well-lit room at the neurological clinic of the university hospital.

The Ethics and Research Committee of the Pontifical Catholic University of Campinas approved the study. The participants were informed of the research protocol and signed the informed consent form.

### Procedures

Data collection was performed during the outpatient care of patients, and the following variables were investigated:

- Demographic and clinical variables: Data on age of onset, type and frequency of seizures, and number of ASMs in use were collected. Electroencephalography (EEG) and magnetic resonance imaging (MRI) data were used to characterize the epileptic syndrome;
- Habitual physical activity questionnaire (HPAQ): The questionnaire was applied to measure habitual physical activity, using Likert Scale, with 16 items that assess the patterns of PA intensity, frequency, and length in the last 12 months and involve three usual contexts, i.e., occupational PA, PA in sport, and PA during leisure time, and the total score. Higher scores correspond to a more frequent practice of PA. The questionnaire was culturally adapted to Brazil^15^;
- Satisfaction scale for physical activity (SSPA): A six-question, Likert-type scale (0=no, 1=a little, 2=a lot) was applied, composed of two dimensions, i.e., satisfaction with the practice of PA when walking and satisfaction with the practice of PA with moderate/vigorous intensity, and the total score. Higher scores indicate greater satisfaction with the practice. The scale was translated and adapted to the Brazilian context^16^;
- Quality of Life in Epilepsy Inventory (QOLIE-31): An epilepsy-specific QoL inventory was used. The total score ranges from 1 to 100, and higher scores indicate a better QoL. This instrument was validated in Brazil^17^.

Cognitive assessment was performed with the application of:

- Mini-Mental State Examination (MMSE)^18^: it is a cognitive screening test. The maximum score is 30 points;
- Brief Cognitive Battery-Edu (BCB-Edu)^19^: It was used to assess cognitive performance. The following are assessed: naming; incidental memory; immediate memory; learning; delayed recall; recognition. The SVF test (animals in one minute) and the clock drawing test were also applied. For immediate memory, learning, delayed recall, and recognition, the cut-off scores were <five; <seven; <six; and <nine, respectively; for the SVF test, the cut-off score was 9 for illiterate individuals and for individuals with <eight years of formal education; and for individuals with >eight years of formal education, it was 13.

### Statistical analysis

Exploratory data analysis was performed with the calculation of frequencies and percentages. The Pearson correlation coefficient (r) was used to assess the correlation between data related to the frequent practice of PA with clinical, demographic, and cognition variables and with QOLIE–31 and SSPA scores.

Based on the significant correlations, stepwise linear regressions were performed to calculate which clinical aspects (age, age of onset, type of epileptic syndrome, and length of epilepsy), and cognitive aspects associated with the HPAQ (occupational PA, PA in sport, PA in leisure time, and total score). The best models were selected based on their p-values (p).

The data were treated by the IBM Statistical Package for Social Sciences (SPSS) software, version 22. The significance level was set to 5% (p<0.05).

## RESULTS

The sample consisted of 60 PWEs and 30 individuals were included in the composition of the CG. Among the PWEs, 40 (66.7%) were employed, 13 (21.7%) were unemployed, and 7 (11.7%) were retired or students; 35 (58.3%) patients were in a stable relationship and 25 (41.7%) patients were single or divorced.

There was no difference in age, sex, and educational level between PWEs and CG individuals. Frequency and satisfaction with PA scores were significantly lower in the PWEs when compared to CG subjects. Cognitive performance in most tests was lower in the PWEs, compared to the CG (Table 1).

**Table 1:** Demographic and clinical data and scores in the cognitive assessment and physical activity scales of the people with epilepsy and control-group individuals.

		PWEs (n=60)	CG (n=30)	p
Age, years (SD)		42.4 (±13.6)	40.3 (±10.5)	0.415*
Sex: female/male		30/30	15/15	1.000†
Educational level, years (SD)		7.1 (±4.0)	6.6 (±2.6)	0.486*
Age of onset, years (SD)		19.5 (±13.9)		
Length of epilepsy, years (SD)		22.9 (±14.7)		
QOLIE-31, score total (SD)		68.8 (±17.4)		
Seizure frequency in the past year	≥once/month; 1–11 times/year; <once/year	22/10/28		
Seizure type	Focal/generalized	34/26		
Number of ASMs	1/≥2	41/19		
Epileptic syndrome: focal	Unknown etiology/structural	19/41		
QOLIE-31, score total (SD)		68.8 (±17.4)		
MMSE, score total (SD)		24.7 (±4.0)	25.1 (±2.3)	0.608*
BCB-Edu, score (SD)	Naming	9.6 (±1.0)	9.9 (±0.1)	0.333*
	Incidental memory	5.8 (±1.8)	6.2 (±1.4)	0.472*
	Immediate memory	7.2 (±1.9)	7.5 (±1.9)	0.152*
	Learning	7.7 (±1.9)	8.1 (±1.4)	0.012* ‡
	Delay recall	6.7 (±2.1)	8.3 (±1.5)	<0.001* ‡
	Recognition	7.9 (±2.1)	9.9 (±0.5)	0.038* ‡
SVF, score (SD)		9.1 (±1.6)	14.0 (±3.9)	0.013* ‡
Clock drawing test, score (SD)		5.6 (±2.8)	5.6 (±2.8)	0.427*
HPAQ, score (SD)	Occupational PA	2.0 (±1.4)	3.3 (±0.4)	<0.001* ‡
	PA in sport	1.7 (±1.2)	3.0 (±0.7)	0.003* ‡
	PA in leisure time	1.7 (±1.1)	2.3 (±0.6)	0.075*
	Total score	5.9 (±3.0)	8.9 (±1.4)	<0.001* ‡
SSPA, score (SD)	The individual regularly walks	4.43 (±2.18)	5.0 (±1.5)	0.154*
	PA with moderate and vigorous intensity	2.88 (±2.66)	4.53 (±1.67)	0.003* ‡
	Total score	7.32 (±4.19)	9.53 (±2.76)	0.001* ‡

Abbreviations: PWEs: people with epilepsy; CG: control group; SD: standard deviation; QOLIE-31: Quality of Life in Epilepsy Inventory; ASMs: antiseizure medications; MMSE: Mini-Mental State Examination; BCB-Edu: Brief Cognitive Battery-Edu; SVF: Semantic Verbal Fluency Test; HPAQ: Habitual Physical Activity Questionnaire; PA: physical activity; SSPA: Satisfaction Scale for Physical Activity.

Notes:

* *t* test;

† chi-square;

‡ p<0.05.

### Patients with epilepsy: demographic and clinical aspects, quality of life, satisfaction scale for physical activity, habitual physical activity questionnaire

Age was negatively correlated with PA in leisure time. Educational level was correlated with higher PA in sport (Table 2). There was no difference in HPAQ scores according to sex and marital status. In the comparison among employed, unemployed, or retired/students, it was observed that employed individuals have significantly higher scores in the occupational PA context (Kruskal-Wallis test; 2.8±1.1 vs 1.2±1.6 vs 1.9±0.4; p=0.001); on PA in leisure (2.4±0.9 vs 2.3±1.0 vs 1.1±0.2; p=0.003); and on the total HPAQ score (7.6±2.0 vs 5.9±2.1 vs 4.8 ±0.7; p=0.001). There was no significant difference in the PA in sport context of the HPAQ.

**Table 2:** Correlation between the Habitual Physical Activity Questionnaire, clinical and cognitive aspects, and the Satisfaction Scale for Physical Activity and Quality of Life in Epilepsy Inventory-31 scores in patients with epilepsy.

		HPAQ			
		Occupational	Sport	Leisure time	Score total
		r	r	r	r
Age		0.003	-0.179	-0.290*	-0.226
Educational level		0.097	0.291*	0.064	0.250
Age of onset		-0.032	0.118	-0.137	-0.024
Length of seizure disorder		0.800	-0.276*	-0.134	-0.185
SSPA	Walking	0.144	0.406†	0.115	0.334†
	Moderate and vigorous	0100	0.312*	0.100	0.278*
	Total score	0.138	0.410†	0.124	0.351*
MMSE		0.327*	0,131	-0.072	0.254*
Identification		0.300*	-0.026	-0.078	0.149
Naming		0.085	-0.108	0.138	0.057
Incidental memory		0.047	0.057	0.046	0.069
Immediate memory		0.381†	-0.100	0.001	0.188
Delayed recall		0.096	-0.082	0.064	0.065
Recognition		-0.058	0.073	0.123	0.067
SVF test		0.427†	0.108	0.029	0.336†
Clock drawing test		-0.021	0.081	0.113	0.095
QOLIE-31 (total score)		0.198	-0.081	0.116	0.136

Abbreviations: HPAQ: Habitual Physical Activity Questionnaire; SSPA: Satisfaction Scale for Physical Activity; MMSE: Mini-Mental State Examination; SVF: Semantic Verbal Fluency; QOLIE-31: Quality of Life in Epilepsy Inventory; r: Pearson’s correlation coefficient.

Notes:

* p<0.05;

† p<0.001.

Longer length of epilepsy correlated with lower PA in sport. There was no difference in HPAQ scores according to the type and frequency of seizures, the number of ASMs used, the epileptic syndrome, and age of onset.

Higher scores on the HPAQ (PA in sport and total score) correlated with higher scores on the SSPA (total and walking scores, and in moderate and vigorous PA).

There was no correlation between the total score on the QOLIE-31 and the scores on the HPAQ and the SSPA.

In the linear regression model, the significant predictive factor associated with habitual PA practice (total score and occupational PA) was the SVF test. The only significant predictive factor associated with the practice of PA in sport was the length of epilepsy. Age was the only significant predictor associated with habitual PA practice in leisure time. The other aspects were excluded from the equation (Table 3). The explanatory power of this model (R2) was low, which may indicate that other unmeasured variables may be related and may partially explain the data obtained.

**Table 3:** Linear regression to assess clinical and cognitive factors associated with the practice of physical activity in the people with epilepsy.

		Variables in the equation	Beta coefficient	SE	t	p
HPAQ	Total score*	Semantic verbal fluency test	0.207	0.075	2.766	0.009
	Occupational†	Semantic verbal fluency test	0.164	0.046	3.534	0.001
	Sport‡	Duration of epilepsy	-0.021	0.010	-2.011	0.049
	Leisure time§	Age	-0.019	0.009	-2.179	0.034

Abbreviations: HPAQ: Habitual Physical Activity Questionnaire; SE: standard error. Notes: Nagelkerke’s R^2^;

* R^2^= 118;

† R^2^=427;

‡ R^2^=262;

§ R^2^=282.

## DISCUSSION

This study assessed PWEs treated as outpatients with a mean age of 42.4 years and a mean time of epilepsy of 26.1 years. It was observed that PWEs practice less PA in intensity and frequency and perceive less satisfaction with this practice than CG individuals. Similar to our findings, studies described that despite evidence of significant benefits of PA, PWEs have a more sedentary lifestyle or are inactive or do not practice PA at the recommended intensity and regularity, as well as they do not perceive satisfaction with their practice^1–4^.

When assessing 97 PWEs, Bem et al., described lower PA practice in epilepsy, and reinforced that PA practice is related to aspects of work and employability, and that the differences in literature data occur due to socioeconomic and cultural differences analyzed samples^20^. In a recent review study, 54.5% of the papers found lower PA levels and lower propensity to be involved in PA in epilepsy; however, in the other studies, no significant differences were found between PWEs and CG individuals^21^.

### Patients with epilepsy: relationship between physical activity with demographic and clinical aspects and quality of life

The practice of PA was significantly higher in younger individuals, in those with higher formal education and in the employed, but with no difference between sexes and marital status. The longer length of epilepsy correlated with the lower practice of PA in leisure time, which could suggest that lower adherence to PA is possibly associated with the fear of practicing PA in inducing or worsening seizures or even accidents, due to prejudice or overprotection^1,22^.

Recent studies pointed out the practice of exercises as a therapeutic strategy in epilepsy, as for example, in a literature review study^23^ that investigated the benefits, side effects, and impact of PA in PWEs. In this review, the authors showed that PA is beneficial and safe for PWEs, further suggesting better seizure control, psychosocial benefits, and improvement in comorbidities^23^ and there is evidence suggesting that PA and active participation in sports could contribute to seizure control, in addition to producing broader psychosocial and health benefits^24^.

A greater frequency of PA was positively correlated with greater satisfaction with its practice, suggesting that perceived satisfaction seems to be a mediator of PA practice and should be considered for long-term effectiveness in health care and intervention.

Surprisingly, there was no correlation between the practice of PA and the perception of QoL, which may suggest that they are different constructs, or even the perception of stigma associated with epilepsy in PWEs. Differently, other studies associated a better well-being in epilepsy with greater social integration and the practice of PA, with few exceptions^1,4,5^.

Unlike the findings found in the present study, in a paper recently conducted in Texas assessed the impact of PA and medication adherence, on seizure frequency and QoL of PWEs. The authors showed that PA was positively associated with QoL and negatively associated with seizure frequency for PWEs, further suggesting that physically active PWEs tend to have fewer seizures and a better QoL^24^.

### Physical activity and cognitive aspects

As already discussed in the literature, some PWEs have worse cognitive performance, particularly memory-related, when compared to CG-group individuals, and these deficits significantly contribute to the impairment of lifestyle and QoL in epilepsy^25,26^.

A greater practice of PA was significantly associated with better performance in perception, immediate memory, SVF, and in the MMSE, in the univariate analysis. In linear regression, it was observed that greater PA practice in different contexts is associated with cognitive, clinical, and demographic aspects, suggesting a positive relationship between PA and cognition in epilepsy. The effects of PA on cognitive function in PWEs were demonstrated in a randomized controlled trial, which investigated the effect of a twelve-week exercise program in PWEs and control groups. The study provided evidence that combined physical training improves executive function in PWEs, showing major improvements in attention and language-related tasks^27^. This evidence and the findings in the present study may suggest the relevance of PA in cognitive function in epilepsy.

These aspects emphasize the importance of deepening the assessment of the PA’s contribution to cognition in PWEs as a strategy to lessen the negative effect of epilepsy on cognitive function.

This study has some limitations; its design was cross-sectional, and the sample consisted of patients assisted at a single university hospital. The study used validated instruments, and in the literature, there are few papers that assess the relationship between PA and cognition in epilepsy. However, the severity of the disease may compromise data generalization.

In conclusion, PWEs practice less PA in intensity and frequency and perceive less satisfaction with this practice than CG individuals.
